# Artificial intelligence in hospitals: Legal uncertainties and emerging risks for patient safety

**DOI:** 10.17179/excli2025-8679

**Published:** 2025-07-17

**Authors:** Meriem Gaddas, Mohamed Ben Dhiab, Imen Ben Saida, Helmi Ben Saad

**Affiliations:** 1 University of Sousse, Faculty of Medicine 'Ibn el Jazzar' of Sousse, Farhat HACHED University Hospital, Research Laboratory LR12SP09 'Heart Failure' Sousse, Tunisia; 2 Department of Physiology and Functional Explorations, Farhat HACHED University Hospital, Sousse, Tunisia; 3 University of Sousse, Faculty of Medicine of Sousse, Department of Forensic Medicine, EPS Farhat HACHED of Sousse, Tunisia; 4 University of Sousse, Faculty of Medicine of Sousse, Department of Intensive care, Farhat Hached University Hospital, Sousse, Tunisia

## ⁯⁯⁯⁯⁯

Thanks to its promises of performance and safety, artificial intelligence (AI) has become a pervasive technology in modern life, influencing nearly every facet of human activity (Ganesh, 2020[9]). Its rapidly expanding impact affects hundreds of millions of individuals worldwide and is profoundly reshaping the structures of our societies (Ganesh, 2020[9]). However, a lesser-known and rarely discussed side of this technology involves serious failures-some of which have had fatal consequences, such as airplane crashes and automated vehicle accidents (Ganesh, 2020[9]). Despite its undeniable contribution to the optimization of healthcare delivery, the integration of AI into hospital settings is not without risks (Becker's Health IT, 2024[1]; Boussina et al., 2024[4]; Dufour et al., 2020[8]; henricodolfing, 2024[10]; Luxton, 2019[11]; Obermeyer et al., 2019[13]; Powles and Hodson, 2017[14]; Schertz et al., 2023[15]; Wong et al., 2021[17]; Zhou et al., 2019[18]) (Supplementary information, Table S1). AI systems have already exhibited failures that have resulted in patient harm (Table S1). These incidents raise critical concerns regarding patient safety and the legal accountability associated with the use of such technologies (Table S1). Hospitals, like other healthcare providers, increasingly promote the integration of AI technologies and may therefore be held liable in the event of accidents or patient harm (Solaiman and Malik, 2025[16]). This liability may stem from their institutional duty to ensure that AI systems are properly validated, maintained, and used in accordance with applicable regulatory requirements. 

With an emphasis on responsibility, regulatory loopholes, and the consequences for clinical practice and patient safety, this editorial sought to critically examine the medico-legal issues and safety concerns related to the integration of AI systems in healthcare. Hospitals are also responsible for providing adequate training to healthcare professionals on the appropriate use of AI systems and for implementing safeguards to prevent errors or algorithmic biases that could compromise clinical decision (Duffourc and Gerke, 2023[7]). Physicians, in turn, may face allegations of medical malpractice if the use-or misuse-of AI is deemed to fall below the accepted standard of care (Cohen et al., 2024[5]). Furthermore, the integration of AI raises concerns regarding informed consent, particularly when patients are unaware of the role AI plays in their diagnostic or therapeutic management (Duffourc and Gerke, 2023[7]).

The concept of medical liability lies at the heart of current legal reforms addressing the use of AI in healthcare (Duffourc and Gerke, 2023[7]; Solaiman and Malik, 2025[16]). Notably, the European Union has introduced two major legislative instruments (Duffourc and Gerke, 2023[7]): *(i)* the AI act, which is based on the classification of medical AI systems as high-risk, emphasizing transparency and the preservation of human oversight (Solaiman and Malik, 2025[16]), and *(ii)* the AI liability directive, which addresses the issue of accountability for harm caused by AI, by establishing new rules that make it easier for individuals to seek compensation (Duffourc and Gerke, 2023[7]). However, it is worth noting that in 2020, the European Parliament rejected the proposal to grant AI systems a form of 'electronic legal personality,' arguing that such a status would undermine fundamental principles of law and risk diluting human accountability, thereby weakening legal protections for victims (Duffourc and Gerke, 2023[7]). Consequently, AI remains legally defined as a 'product' meaning that developers may be held liable for system failures that result in patient harm (Duffourc and Gerke, 2023[7]). In the United States, the regulation of AI in healthcare is primarily governed by federal laws focused on safety, such as those enforced by the food and drug administration (Cohen et al., 2024[5]). Liability in the event of an adverse outcome falls under civil law; however, assigning responsibility remains complex due to the involvement of multiple stakeholders and the inherently opaque nature of AI systems (Cohen et al., 2024[5]).

Despite recent (*ie*; after 2020) legal reforms, it is important to note that, to date (*ie*; early July 2025), no AI system has been prosecuted or held legally accountable for harm before any national or international court (Solaiman and Malik, 2025[16]). Although AI is increasingly used in high-stakes sectors such as healthcare, transportation, and justice, the law continues to treat it as a tool rather than as a legal subject (Solaiman and Malik, 2025[16]). Moreover, there is currently no specific regulatory framework governing the liability of the various actors involved in the AI supply chain, nor are there harmonized operational guidelines to ensure the safe and ethical integration of AI technologies into clinical practice-particularly with respect to external validation, algorithmic decision traceability, and alignment with existing medical device regulations (Maliha et al., 2021[12]). 

Current (*ie*, early July 2025) legal frameworks are often ill-equipped to address the complexities introduced by AI systems, resulting in legal gaps that may be exploited to evade liability. These shortcomings stem primarily from at least the following seven factors:

1. *Lack of legal status*: AI has no recognized legal personality, meaning it cannot be sued or insured, nor can it hold assets to compensate victims. In legal proceedings, fault must therefore be transferred to a human actor or a legal entity such as a corporation (Bertolini and Episcopo, 2022[2]).
2. *Absence of clear attribution of liability*: Traditional legal doctrines are not designed to hold non-human agents accountable. AI systems lack intent, moral agency, or autonomous legal will, all of which are central to the attribution of fault under current liability models (Bertolini and Episcopo, 2022[2]).
3. *Multiplicity of actors and fragmented responsibility*: AI development and deployment often involve a diffuse network of stakeholders-including developers, healthcare institutions, and clinicians-making it difficult to trace failures and assign responsibility (Maliha et al., 2021[12]).
4. *Unpredictability of AI behavior*: Legal liability frameworks are generally grounded in the foreseeability of actions (*eg*; negligence or intent) (Bottomley and Thaldar, 2023[3]). However, the unpredictable nature of many AI systems challenges this premise and raises significant safety concerns in clinical care. This unpredictability is attributable to two elements. The first is related to the 'black box' nature of many machine-learning models, where the decision-making process is opaque and often not interpretable by users. This lack of transparency complicates the establishment of causal links between algorithmic faults and patient harm and hinders technical or design flaw analysis (Duffourc and Gerke, 2023[7]). The second element concerns the system's relative autonomy and capacity for evolution through self-learning. AI can adapt its decision-making criteria over time-sometimes without explicit human oversight-leading to unanticipated outputs that were not originally programmed (Duffourc and Gerke, 2023[7]). In many cases, harmful autonomous behavior becomes evident only after the system has been deployed, further complicating the traceability of the initial failure and legally diluting responsibility across physicians, manufacturers, and the algorithm itself (Duffourc and Gerke, 2023[7]).
5. *Data quality*: The accuracy and completeness of training data significantly influence AI performance. For instance, datasets that underrepresent certain patient groups can lead to suboptimal clinical decisions and racially biased outcomes (Cross et al., 2024[6]).
6. *Inherent complexity and stochastic nature of medical data*: Unlike structured datasets (*eg*; mathematical data), medical data are heterogeneous and probabilistic, making it difficult to ensure consistent and reliable AI performance (Table S1).
7. *Lack of human oversight due to automation bias*: Excessive reliance on AI may lead to clinician deskilling and diminished critical engagement, undermining secondary human control and increasing the risk of error propagation (Table S1). AI systems should augment-rather than replace-clinical judgment. To navigate the technological complexity and mitigate automation bias, continuous human oversight and close collaboration among all stakeholders are essential. Above all, patient safety must remain the foremost priority.

In conclusion, even though AI has a lot of potential to improve clinical judgment and healthcare delivery, its use is troubled by ethical, legal, and technological issues that the current regulatory frameworks are unable to appropriately handle. Efforts to protect patient rights and maintain therapeutic accountability are complicated by the unclear attribution of liability, lack of transparency, and unpredictable nature of AI conduct. Establishing a strong legal framework that outlines roles for all parties involved, guarantees algorithmic openness, and prioritizes patient safety is necessary for the safe and moral integration of AI into medical practice.


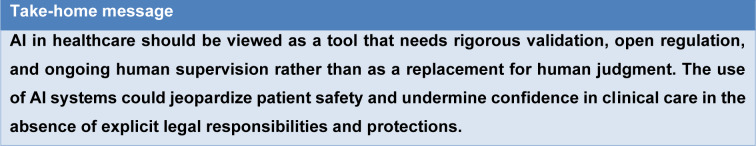


## Declaration

### Artificial Intelligence

The authors would like to reveal that in order to improve the consistency and clarity of the manuscript authoring, artificial intelligence techniques like QuillBot and ChatGPT ephemeral were used. Without changing the scientific substance or creating any new material, the tools were used solely for language improvement, making sure the text was understandable and cohesive.

### Authors' contributions

All authors: Literature search, Manuscript preparation and Review of manuscript. All authors read and approved the final manuscript.

### Conflict of interest

The authors confirm that there is no conflict of interest.

### Data availability statement

Data sharing not applicable to this article as no datasets were generated or analyzed during the current study.

## Supplementary Material

Supplementary information
