# Supplementary material for: Artificial intelligence in hospitals: Legal uncertainties and emerging risks for patient safety
Source: EXCLI J. 2025 Jul 17;24:824–7. doi: 10.17179/excli2025-8679 (PMC12835522; doi:10.17179/excli2025-8679)
Supplement: Supplementary information [file EXCLI-24-824-s-001.pdf]

EXCLI Journal 2025;24:824-827 – ISSN 1611-2156
Received: June 23, 2025, accepted: June 28, 2025, published: July 17, 2025

Supplementary information to:

Letter to the editor:

ARTIFICIAL INTELLIGENCE IN HOSPITALS: LEGAL UNCERTAINTIES AND EMERGING RISKS FOR PATIENT SAFETY

Meriem Gaddas[1,2], Mohamed Ben Dhiab[3], Imen Ben Saida[4], Helmi Ben Saad[1,2]

1 University of Sousse, Faculty of Medicine 'Ibn el Jazzar' of Sousse, Farhat HACHED University Hospital, Research Laboratory LR12SP09 'Heart Failure' Sousse, Tunisia
2 Department of Physiology and Functional Explorations, Farhat HACHED University Hospital, Sousse, Tunisia
3 University of Sousse, Faculty of Medicine of Sousse, Department of Forensic Medicine, EPS Farhat HACHED of Sousse, Tunisia
4 University of Sousse, Faculty of Medicine of Sousse, Department of Intensive care, Farhat Hached University Hospital, Sousse, Tunisia

* **Corresponding author:** Helmi Ben Saad, University of Sousse, Faculty of Medicine 'Ibn el Jazzar' of Sousse, Farhat HACHED University Hospital, Research Laboratory LR12SP09 'Heart Failure' Sousse, Tunisia. E-mail: helmi.bensaad@rns.tn

https://dx.doi.org/10.17179/excli2025-8679

This is an Open Access article distributed under the terms of the Creative Commons Attribution License (https://creativecommons.org/licenses/by/4.0/).

Table S1: Major documented cases of failures involving artificial intelligence (AI) tools already deployed in clinical practice

**Year, references**	**Software type Developer**	**Nature of failure**	**Patient harm**	**Charge/Lawsuit Outcome of the case**	**Responsibility attribution gaps**
**2013-2021** (henricodolfing, 2024; Luxton, 2019; Zhou et al., 2019)	**WFO:** diagnostic and therapeutic decision support .applied in 14 countries<br>IBM & Memorial Sloan Kettering cancer center	High discordance rates compared to recommendations, varying depending on the histological type (which can reach up to 88 %)<br>Suggestions for inappropriate and even dangerous treatments (*eg*; unjustified aggressive chemotherapy)	Inappropriate treatments, management errors	No known direct legal action<br>IBM disabled Watson in 2021<br>Reputation tarnished	Lack of transparency<br>Black box<br>Untraceable recommendations
**2015-2021** (Powles and Hodson, 2017)	**DeepMind Health/Streams** (Follow-up of kidney failure cases)<br>Google (DeepMind) plus NHS (UK's NHS)	Illegal use of patient data without their explicit consent (data privacy issue) .Errors in clinical notifications	Delays in the detection of kidney failures<br>No directly proven deaths, but identified risks and documented cases	UK authorities' investigation for violation of the data protection act (1998)<br>No prior approval from authorities<br>Administrative sanctions, withdrawal of the initial version (but the emergence of other derivative applications)<br>A public mistrust of this technology	The AI nature of the tool not recognized by the developers<br>Shared responsibility between the developer and the NHS
**2019** (Obermeyer et al., 2019)	Population health risk stratification algorithm<br>Optum (a subsidiary of United Health)	Structural racial bias: The algorithm prioritized patients with the highest medical expenses as an indicator of the severity of their health condition<br>The algorithm systematically underestimated the needs of Black patients (with limited income)	The tool underestimated the level of risk and excluded Black patients from necessary intensive or preventive care programs, potentially worsening their health condition	No known individual legal complaints<br>The developer acknowledged the bias and announced a revision of its algorithm<br>No official public sanctions	Lack of a clear regulatory framework on the responsibility for algorithmic biases<br>Opacity of proprietary algorithms<br>Difficulty in demonstrating

					direct individual harm
**2020** (Dufour et al., 2020)	**Autonomous intensive care ventilators (Evita V300/V500) Dräger Medical**	Automatic activation of a pressure-limiting algorithm without warning	**Resulting in desaturation followed by asystole or coma in intubated patients**, requiring resuscitation<br>Other similar incidents have also been reported	Multiple reports were submitted to the ANSM.<br>Due to the lack of confirmed technical malfunction, the cases were closed without resolution, no legal action was taken<br>At the request of ANSM, the manufacturer was initially required to update the user manual and later committed to "correcting" the operating algorithms to enhance patient safety	Concerns persist regarding lack of transparency<br>Non-traceable recommendations<br>Presence of a "black box" system<br>No formal investigations were conducted for the reported cases
**2021** (Wong et al., 2021)	ESM: Early detection of sepsis<br>**Deployed in hundreds of hospitals across the US**<br>Epic systems corporation (largest health care software vendors in the world)	Low diagnostic performance<br>Sensitivity of 33 % and specificity of 83 %<br>Generates a high volume of alerts, leading to alert fatigue and potential patient safety risks<br>**Poor calibration**: inconsistency between predicted scores and actual patient risk	67 % of patients with sepsis were not identified by the model<br>7 % of patients did not receive timely antibiotic treatment	No specific cases have been documented<br>No legal actions have been initiated<br>2022: In response to growing criticism, Epic made adjustments to the model<br>A 2023 study showed that ESM still underperformed compared to other available tools (Schertz et al., 2023)<br>In light of ESM's limitations, new AI-based models have been developed, such as the COMPOSER model (Boussina et al., 2024)<br>Despite its flaws, ESM continues to be used in hundreds of US hospitals (Becker Health IT, 2024)	Implemented without prior external validation

ANSM: French national agency for the safety of medicines and health products; **ESM**: Epic sepsis model; **IBM**: International business machines corporation; **NHS**: National health service. **WFO:** Watson for oncology
